# Information needs and engagement profiles of adults undergoing ambulatory surgery: a qualitative study

**DOI:** 10.1016/j.ijnsa.2026.100703

**Published:** 2026-10-09

**Authors:** Wen Qin, Qin Zhu, Yang Shen, Xiaoyun Dai, Yi Xu, Yingying Huang, Yao Tian

**Affiliations:** a Affiliated Hospital of Nantong University, No 20 Xisi Road, Nantong, Jiangsu, 226001, PR China; b College of Nursing and Rehabilitation, Nantong University, Nantong, Jiangsu, 226001, China

**Keywords:** Ambulatory surgical procedures, Patient education as topic, Qualitative research, Perioperative nursing, Patient participation, Information seeking behavior

## Abstract

**Background:**

Ambulatory surgery compresses preparation, treatment and discharge into a short period, followed by recovery at home. Information support must address emotional concerns, information preferences and ways of engaging with care. Although unmet information needs have been described, less is known about how these needs form recognisable engagement profiles.

**Objective:**

To explore adult patients’ information needs across the ambulatory surgery pathway and develop empirically informed engagement profiles to guide future personalised information support, this qualitative descriptive study recruited thirty-three adult patients through purposive maximum-variation sampling at an ambulatory surgery centre at a tertiary hospital in Eastern China from October to December 2025; nine caregivers of children interviewed in the original study were excluded from this analysis.

**Methods:**

Individual semi-structured interviews were audio-recorded and transcribed verbatim. Thematic analysis identified information domains, followed by cross-case synthesis to construct exploratory engagement profiles from adult patient accounts.

**Results:**

Six domains concerned disease and diagnosis, surgical procedures, anaesthesia, post-discharge care and follow-up, psychosocial and practical support, and information delivery preferences. Four non-exclusive profiles were constructed: Anxious Proceduralist, Guidance-Seeking User, Collaborative Learner and Pragmatic Self-Manager. These profiles describe differing preferences for procedural detail, clear guidance, dialogue and practical recovery support.

**Conclusions:**

Similar information needs may be organised through different ways of engaging with care. The profiles offer preliminary, hypothesis-generating design tools for personalised information support. Refinement, item development, co-design and feasibility testing are required before clinical use. The findings concern adult patients and cannot be transferred directly to caregivers of children.

**Registration:**

Not registered.

**What is already known**

• Ambulatory surgery patients report unmet needs for information about procedures, anaesthesia, recovery and follow-up, alongside differing emotional concerns and communication preferences.
• Personas can inform person-centred health design, but their application to ambulatory surgery education remains underdeveloped.

**What this paper adds**

• Six familiar information domains are linked to differing adult patient engagement preferences rather than a single pattern of information use.
• Four non-exclusive engagement profiles connect information needs with coping orientation, participation in decisions and delivery preferences.
• The profiles identify candidate features for future assessment, co-design and feasibility testing of personalised perioperative information support.

## 1 Introduction

Ambulatory surgery involves admission, surgery and discharge on the same working day and has become an important model of surgical care (Bailey et al., 2019). This compressed pathway also restricts opportunities for communication. During brief clinical encounters, patients must understand preparation, surgical procedures, anaesthesia, discharge instructions and recovery at home, often while experiencing uncertainty and anxiety (Deng et al., 2019). A meta-ethnography similarly identified a need for better information to prepare patients for day surgery and recovery (Thoen et al., 2024).

Needs-based preoperative education reduced anxiety and improved satisfaction in a randomised trial involving day surgery patients (Wongkietkachorn et al., 2018). However, the information provided may not match patients’ concerns (Alacadag and Cilingir, 2018). The problem is therefore more than a shortage of information. Standard leaflets, brief verbal instructions and generic online materials may convey accurate content without accommodating differences in anxiety, understanding, decision preferences or desired level of detail.

Digital education and communication platforms offer opportunities to extend support beyond the hospital through repeat access to instructions, reminders and recovery information. Their usefulness depends on how well content and presentation match the individual’s situation. Social cognitive theory emphasises support for self-efficacy, while self-determination theory highlights autonomy, competence and relatedness (Bandura, 2004; Ng et al., 2012). Attention to information-seeking preferences and accessibility may help translate these principles into education design (Miller, 1987; Kim and Xie, 2017). These theoretical perspectives inform interpretation; they do not establish participants’ psychological traits or health literacy.

Personas offer a way to connect qualitative evidence with intervention design. They are empirically informed representations of recurring goals, behaviours, constraints and preferences, intended to make meaningful variation visible rather than rigidly classify individuals (Bhattacharyya et al., 2019; Vosbergen et al., 2015). Recent applications address psychosocial needs, perinatal mental health and disadvantage in digital access (Perego et al., 2024; Zimmermann et al., 2024; Husain et al., 2024). Yang et al. (2025), for example, developed five self-management personas for people undertaking home-based cardiac rehabilitation. In ambulatory surgery, this approach may help identify how to provide relevant information at closely spaced points of care, as responsibility for recovery moves rapidly to patients and families.

Previous studies have identified common perioperative information topics and evaluated educational interventions. Less attention has been paid to how these needs combine into recognisable engagement profiles that might inform more individualised education. This study therefore explored information needs across the ambulatory surgery pathway among adult patients and constructed empirically informed profiles describing how they engage with perioperative information. It provides a qualitative foundation for subsequent design and evaluation; it does not test a digital intervention.

## 2 Methods

### 2.1 Study design and reporting

A qualitative descriptive design was used to develop a detailed, practice-oriented account of participants’ experiences and information needs (Sandelowski, 2000). Reporting was guided by the Standards for Reporting Qualitative Research (O’Brien et al., 2014). The completed reporting checklist identifies both reported items and remaining gaps.

### 2.2 Setting

The study took place from October to December 2025 at the ambulatory surgery centre of a large tertiary teaching hospital in Jiangsu Province, Eastern China. The centre performs >8000 ambulatory procedures annually across several specialties. Patients generally complete preoperative assessment before admission, undergo surgery on the day of admission and return home once clinical discharge criteria are met.

### 2.3 Participants and sampling

Purposive maximum-variation sampling sought diversity in age, gender, education and surgical specialty. Eligible participants were adults aged 18 years or older who were scheduled for ambulatory surgery, could communicate fluently in Mandarin and were willing to provide written informed consent. Exclusion criteria were cognitive impairment, a diagnosed severe mental illness or emergency surgery.

The original study interviewed 33 adult patients and nine caregivers of children. The present analysis is restricted to the 33 adult patients to distinguish patients’ own information use and self-management from caregiver-mediated paediatric care. The nine caregiver interviews were excluded from the adult thematic analysis and profile synthesis. This restriction concerns analytical focus and does not diminish the value of caregiver perspectives. The original study was not designed to establish a separate, sufficiently developed set of caregiver profiles.

Recruitment in the original study was guided by information power, considering the study aim, sample specificity and quality of dialogue, alongside the absence of new substantive themes in three consecutive interviews (Malterud et al., 2016). This describes the original recruitment process, rather than a separately demonstrated stopping criterion for the adult subset. The present report includes 33 adult patients; independent thematic sufficiency for this subset was not prospectively established.

### 2.4 Data collection

Individual face-to-face semi-structured interviews were conducted in a private consultation room at the ambulatory surgery centre. The interview guide was developed through a focused literature review and consultation with four clinical experts, and was piloted with two day surgery patients.

Interviews explored needs at decision-making before admission, while waiting for surgery, at discharge and during anticipated or early recovery at home, as well as preferred delivery formats. Example questions included: “What were your main questions when you first decided to have surgery?”; “While waiting for surgery, what information did you feel was missing?”; “What are you most uncertain about regarding care at home?”; and “How would you ideally like to receive this information?”. Probes sought examples and clarified meaning, such as “Could you tell me more?” and “What do you mean by ‘rest’?”. Supplementary file A reproduces the topics and example questions reported here, rather than the complete original interview guide.

All interviews were audio-recorded with permission; no video recordings were made. Interviews lasted 30–60 min. Written informed consent was obtained beforehand. Trained researchers transcribed recordings verbatim in Mandarin, checked transcripts against the recordings and removed identifiers before analysis. Field notes were recorded immediately after each interview. Quotations in this report were translated into English for publication.

### 2.5 Data analysis

Thematic analysis followed the six-phase framework of Braun and Clarke (2006), supported by NVivo 14. Two researchers independently read and reread the transcripts, generated initial codes and compared their coding. Differences were discussed in coding meetings and resolved by consensus. Where uncertainty remained, a senior qualitative researcher reviewed the relevant extracts and facilitated agreement. A coding log documented code definitions, codebook revisions and reasons for merging or separating themes.

Profile development followed thematic analysis as a separate cross-case synthesis, informed by persona research (Yang et al., 2025). First, the team constructed a case matrix for the adult patients, summarising age group, education, specialty, principal concerns, expressions of anxiety or uncertainty, information-seeking behaviour, participation in decisions and preferred delivery formats. Second, cases were compared to identify recurring configurations of attributes. Third, preliminary profiles linked these configurations to the six information domains and illustrative extracts. Fourth, the profiles were checked against the adult patient dataset. Finally, two senior nursing researchers who had not undertaken the initial coding reviewed the labels, defining attributes and supporting quotations.

A profile was retained only when the pattern was supported by multiple adult cases and related to more than one information domain. Profiles were interpreted as overlapping descriptions of information engagement, rather than mutually exclusive diagnostic categories or validated screening tools. Age and education were contextual attributes, not rules for assigning a person to a profile.

### 2.6 Rigour and trustworthiness

Credibility was supported through prolonged engagement at the centre, peer debriefing within the research team and member checking with five adult participants in the original study. An audit trail recorded recruitment, interview conduct, coding decisions, theme refinement and profile development. Independent coding and discussion of alternative interpretations supported confirmability. Contextual information about the setting, sample and pathway was provided to help readers assess transferability.

### 2.7 Ethical considerations

The study was conducted in accordance with the Declaration of Helsinki and was approved by the hospital ethics committee of the participating institution (approval number 2023-K172-01; institution name withheld for blinded review). All participants provided written informed consent and were informed that participation was voluntary, that withdrawal would not affect their care, and that quotations would be reported anonymously. Data were de-identified, encrypted, and stored on secure, password-protected servers accessible only to the research team.

## 3 Results

### 3.1 Participant characteristics

The analysis included 33 adult patients aged 25–63 years, with a mean age of 44.1 years (standard deviation 11.0). Eighteen participants were men (54.5%) and 15 were women (45.5%). Education ranged from primary school or less to university or above. Surgical specialties included general surgery, ear, nose and throat surgery, gynaecology, orthopaedics and urology. Table 1 presents the participant characteristics.

**Table 1:** Characteristics of adult patients (n = 33).

Characteristic	Category	Number	Percentage
**Age (years)**	18–39	13	39.4
	40–59	17	51.5
	60 or older	3	9.1
**Gender**	Men	18	54.5
	Women	15	45.5
**Education**	Primary school or less	4	12.1
	Junior secondary school	9	27.3
	High school or technical school	12	36.4
	University or above	8	24.2
**Surgical specialty**	General surgery	12	36.4
	Ear, nose and throat surgery	8	24.2
	Gynaecology	6	18.2
	Orthopaedics	4	12.1
	Urology	3	9.1

Percentages use 33 adult patients as the denominator and may not sum to 100 because of rounding. The 18–39-year category contains participants aged 25–39 years; no participants aged 18–24 years were included. These descriptive data do not establish age-specific engagement profiles.

### 3.2 Core information needs across the ambulatory surgery pathway

Six domains described the information participants wanted before admission, while waiting for surgery, at discharge and during anticipated or early recovery at home.

Disease and diagnosis. Participants wanted basic explanations of the nature and causes of their condition, the reason for surgery, possible alternatives and longer-term expectations. One asked, “Could my vocal cord polyp be related to how much I usually talk? Polyps are usually benign, aren’t they?” (P01, woman, 42 years). Another said, “The doctor said there is a growth in my uterus and that it can be removed by hysteroscopy. These growths are usually benign, aren’t they? Will it be sent for testing then?” (P10, woman, 39 years).

Surgical procedures. Participants wanted to understand the likely timing and sequence of surgery, the environment, staff involved, operative steps, complications and expected outcomes. Uncertainty about timing was a recurrent source of anxiety: “If I knew exactly when I was going to the operating theatre, I would feel less panicked” (P07, man, 54 years). Another participant explained, “I have not dared to eat or drink anything since this morning, and I still do not know when I will have surgery” (P13, man, 41 years).

Anaesthesia. Concerns focused on safety, awareness during surgery, cognitive effects, postoperative pain control and differences between anaesthetic options. One participant asked, “Will my memory get worse after general anaesthesia?” (P05, woman, 48 years). Another said, “The doctor said my operation would be under local anaesthesia, but I am worried it might not work well enough. Could I be put to sleep during the operation?” (P07, man, 54 years). These questions express participants’ concerns, rather than evidence of adverse effects or recommendations about anaesthetic care.

Postdischarge care and follow-up. Participants sought specific guidance on wound care, warning signs, medicines, activity restrictions, diet and follow-up. One described uncertainty about care at home: “The doctor said I could go home after the operation today, but no one has told me what to do at home, or whether I will need more intravenous treatment for inflammation or something like that” (P05, woman, 48 years). Another asked, “What if I bleed heavily after going home? Would I have to come back to hospital? I would actually prefer to stay another night for observation” (P09, woman, 34 years).

Psychosocial and practical support. Beyond biomedical facts, participants described feeling vulnerable while waiting, worries about costs and insurance reimbursement, a need for reassurance and a wish for family members to understand how to help after discharge. One said, “I am from out of town and have very little medical insurance, so I have to pay for everything myself. It is quite a financial strain” (P14, man, 42 years). Another explained, “Although the doctor said it is most likely benign, I am still very worried about the pathology result. I have mixed feelings because my maternal grandmother died of breast cancer” (P08, woman, 25 years).

Information delivery preferences. Brief verbal instructions could be difficult to retain, particularly when participants felt anxious. Participants preferred accessible information that could be revisited, including short videos, checklists, diagrams, photographs, official hospital platforms and opportunities to ask questions. One said, “I wish you had a permanent website or an official social media account where all these materials could be posted, so I could look at them whenever I needed to. The nurses do explain things, but sometimes everything is rushed and I forget” (P13, man, 41 years). Another suggested, “Ideally, the platform could organise information into relevant categories. I do not want to look through lots of unrelated information. It could also have a search function or something like an artificial intelligence conversation, so I could find what I want to know myself” (P05, woman, 48 years).

### 3.3 From information domains to engagement profiles

The six domains describe what participants wanted to know. Cross-case synthesis examined how they engaged with that content. Some sought detailed procedural explanations to reduce anxiety, whereas others preferred a simple sequence of instructions. Some looked to clinicians or family members for direction, while others wanted dialogue or practical self-management tools. These recurring configurations informed four non-exclusive profiles: Anxious Proceduralist, Guidance-Seeking User, Collaborative Learner and Pragmatic Self-Manager (Table 2).

**Table 2:** Engagement profiles linking information needs and ways of engaging with care.

Engagement profile	Defining pattern	Salient needs and preferences	Proposed future support
**Anxious proceduralist**	Seeks control by reducing uncertainty; asks detailed “what if” questions and may repeatedly seek procedural and risk information.	Sequence and waiting times; staff involvement; anaesthetic safety, complications and warning signs. Prefers layered detail, visual timelines and official sources.	Ask about procedural anxiety and desired detail. Offer a timetable, staged explanations, orientation to the operating environment and optional additional information.
**Guidance-seeking user**	Prefers concise, trusted guidance and a manageable sequence of actions.	Clear instructions; medicines and wound care; discharge steps and access to help. Prefers low cognitive load and visual prompts.	Agree on a short care plan, check understanding and involve the adult patient’s chosen supporter with consent. Do not infer health literacy from this preference.
**Collaborative learner**	Seeks dialogue, emotional reassurance and opportunities to confirm understanding with clinicians or peers.	Contextual explanations, shared planning, reassurance and opportunities to ask questions. Prefers interactive communication.	Offer questions and discussion, confirm shared understanding and include family members or peers if the patient wishes.
**Pragmatic self-manager**	Prioritises recovery and autonomy; selectively seeks actionable information relevant to work, activity and everyday life.	Return to activity, symptom thresholds, medicine timing, follow-up tasks and recovery milestones. Prefers concise, task-oriented tools.	Offer recovery tasks, activity guidance, reminders and routes for seeking help, linked to the individual’s agreed recovery plan.

Profiles are non-exclusive and exploratory. Proposed support represents design implications, not interventions delivered or evaluated in this study. Age, gender, education and supporter involvement are not classification criteria. Essential safety information is required across profiles.

The Anxious Proceduralist was illustrated by: “My memory is not very good. The doctor spoke so quickly and listed so many possible risks during the operation. Before I had time to process what he said, I was asked to sign. I felt too embarrassed to ask more questions because he seemed very busy, but I still felt uneasy” (P14, man, 42 years).

The Guidance-Seeking User was illustrated by: “If we each had a printed sheet, that would be better. I could check each item without missing anything. The nurse speaks so quickly that I worry I might not remember” (P06, man, 57 years).

The Collaborative Learner was illustrated by: “I find the information online too formal. I do not understand some of the technical terms, and some of it does not seem trustworthy. I would like the doctor to give professional advice and explain it in language I can understand” (P08, woman, 25 years).

The Pragmatic Self-Manager was illustrated by: “I would like reminders when it is time for suture removal, follow-up and collecting my pathology results” (P07, man, 54 years).

Fig. 1 presents illustrative scenarios linking each profile to a possible form of information support. The profiles are preliminary design tools that may overlap across information domains. The pictured encounters and dialogue were constructed for explanation and are separate from the interview quotations reported above.

**Fig. 1:**
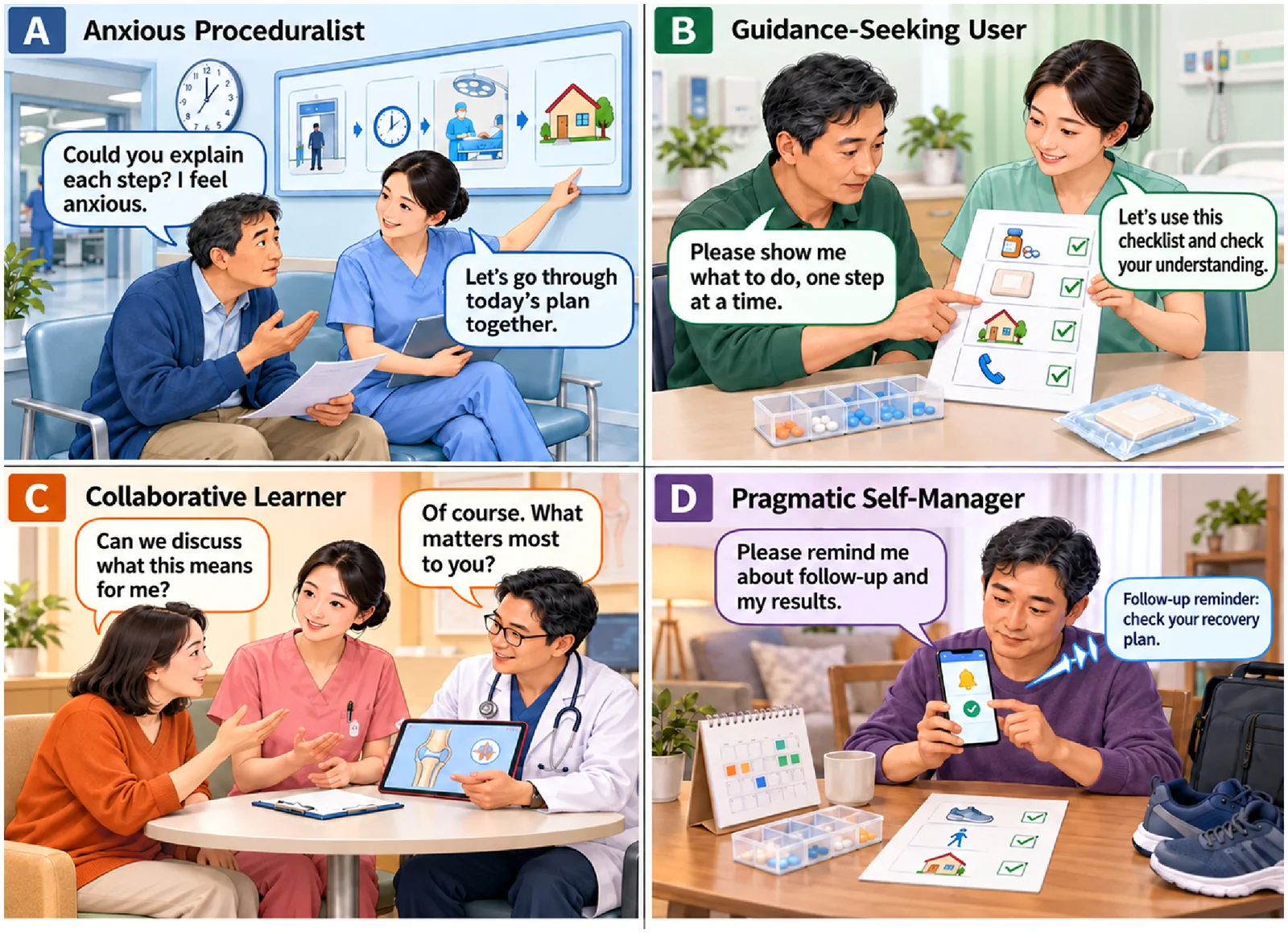
Four engagement profiles and illustrative scenarios of personalised information support for adults undergoing ambulatory surgery.

The four non-exclusive profiles were constructed from interviews with 33 adult patients. (A) Anxious Proceduralist: a patient discusses the day’s sequence with a nurse, illustrating a preference for procedural detail and reassurance about uncertainty. (B) Guidance-Seeking User: a nurse and patient review a checklist, illustrating clear, manageable instructions and checks of understanding. (C) Collaborative Learner: a patient discusses concerns with clinicians, illustrating dialogue, shared interpretation and emotional reassurance. (D) Pragmatic Self-Manager: a patient uses a reminder at home, illustrating practical support for follow-up and recovery tasks. Colours distinguish panels rather than clinical categories. Characters, encounters and speech bubbles are fictional illustrations, not participant portraits, verbatim interview extracts or observations of care. Apparent age and gender do not define the profiles. The proposed support strategies have not been evaluated, and the profiles should not be used for clinical triage without further development and validation.

The profiles do not permanently assign patients to fixed categories. They summarise recurring patterns that may inform future assessment items and tailoring rules. A person’s preferences could change with the stage of care, family support or clinical circumstances; such transitions were not established longitudinally in this study.

## 4 Discussion

This study identified six information domains and four exploratory engagement profiles among adults undergoing ambulatory surgery. The domains are consistent with previous accounts of needs for information about diagnosis, procedures, anaesthesia, recovery, follow-up and reassurance (Thoen et al., 2024). Patients’ accounts of postoperative telephone calls also highlight the value of support and reassurance for self-care at home (Rajala et al., 2025). The present study adds a description of how familiar topics combine with differing coping orientations, decision preferences and preferred ways of receiving information.

### 4.1 Interpretation and contribution of the profile approach

A list of themes indicates that patients want information about anaesthesia or recovery, but provides less guidance about whether they prefer detailed risk explanations, a concise checklist, discussion with a clinician or a goal-oriented recovery plan. The four profiles make these distinctions explicit. They therefore provide a preliminary link between qualitative findings and decisions about the content, timing and channel of patient education.

This contribution is consistent with broader applications of personas in healthcare. Perego et al. (2024) used a human-centred approach to represent psychosocial needs in hereditary angioedema. Zimmermann et al. (2024) developed four personas to support digital intervention design for perinatal mental health, while Husain et al. (2024) examined intersecting disadvantages among marginalised older patients. Yang et al. (2025) described heterogeneous self-management needs in home-based cardiac rehabilitation. These studies provide methodological and design precedents; their profiles do not validate those developed in the present surgical setting.

The Anxious Proceduralist resembles the monitoring orientation described by Miller (1987), in which perceived threat prompts information seeking. This is an interpretive comparison, not a measured psychological classification. Vague reassurance may leave uncertainty unresolved. Layered information could allow patients to choose the amount of detail they receive while supporting confidence in managing care (Bandura, 2004).

The Guidance-Seeking User expresses a preference for clear information with a manageable cognitive load. Such a preference does not establish low health literacy, low confidence or dependence. Plain language, visual prompts and checks of understanding may support accessibility across all profiles (Kim and Xie, 2017). Family or other supporter involvement should reflect the adult patient’s wishes, rather than function as a marker of limited capability.

The Collaborative Learner highlights the relational dimension of perioperative information. Dialogue and reassurance help people interpret what information means for them, consistent with communication approaches that emphasise partnership and trust (Street et al., 2009).

The Pragmatic Self-Manager emphasises actionable tools for resuming daily life and monitoring recovery. This pattern can be interpreted in relation to autonomy and competence in self-determination theory (Ng et al., 2012), without assuming that other participants lack these motivations.

### 4.2 Implications for future personalised education

Translation into practice requires caution. This study did not develop a validated classification instrument or assess usability, feasibility, implementation or clinical effectiveness. Candidate features for a future brief assessment include desired detail, anxiety about uncertainty, confidence with specific instructions, preferred participation in decisions, communication channel and recovery goals. These features should be assessed directly rather than inferred from demographic characteristics.

Future systems could use a profile to suggest an initial pathway that users can modify. For example, a person seeking procedural detail might first see a timetable with optional risk explanations. Someone preferring concise guidance might receive visual instructions and opportunities to explain the plan back in their own words. Collaborative learners might be offered scheduled questions or secure dialogue, while pragmatic self-managers might prefer recovery plans and reminders. A qualitative study of a digital patient-led checklist for cataract surgery likewise supports attention to differing preferences for participation (Stolk-Vos et al., 2018). These are design proposals, not evidence that a particular strategy is effective for a given profile.

Personalisation should also account for diagnosis, planned procedure, adult age group, communication needs and stage of care. Age can provide context but should not determine a profile; this study did not establish age-specific patterns. Before admission, information could address preparation and treatment rationale; during the short waiting period, updates and clarification of immediate concerns may be useful; at discharge, essential care tasks and routes for help require confirmation; and subsequent messages could reinforce the agreed recovery plan. Essential safety information should remain available to every patient, with optional detail and presentation adjusted to preference. More accurate matching of information and any improvements in understanding, anxiety or recovery remain to be tested.

### 4.3 Context and limitations

First, this study was conducted at one tertiary hospital in Eastern China. Local workflows, discharge practices, specialist access, family involvement and use of hospital digital platforms may have shaped the findings. Transferability to smaller hospitals, rural settings, private surgical centres or differently organised health systems requires careful assessment.

Second, the analysis concerns adult patients only. Excluding caregiver interviews avoids conflating patients’ self-management with caregiver-mediated paediatric care, but limits the scope of the conclusions. The nine caregiver interviews in the original study do not establish a separately developed and adequately supported set of caregiver profiles. This is a limitation of the available analysis, not a claim that a fixed sample size determines the quality of qualitative research. Dedicated work should examine caregivers’ own information needs and, where developmentally appropriate, children’s perspectives. Neither the original recruitment stopping criterion nor member checking can be assumed to demonstrate the adequacy of the adult subset independently.

Third, the profiles arose from qualitative synthesis rather than quantitative clustering or psychometric validation. They should not be used for clinical triage or to infer capacity or health literacy.

Fourth, interviews captured anticipated and early recovery needs without longitudinal assessment across recovery. Detailed reporting of interviewer characteristics, prior relationships with participants, interview timing relative to surgery and the process for checking translated quotations remains incomplete, which limits assessment of reflexivity and interpretive transfer. Finally, no digital intervention was tested. Subsequent work should develop assessment items, undertake co-design, evaluate usability and feasibility, and then examine preparation adherence, anxiety, unplanned post-discharge contacts, patient activation and recovery in multicentre studies.

## 5 Conclusions

Ambulatory surgery requires adult patients to manage closely spaced information tasks during brief clinical encounters and the transition home. Information needs involve more than topics to be delivered: they also reflect how people manage uncertainty, participate in decisions, involve others and pursue recovery goals. The four exploratory profiles provide a preliminary framework for these differing forms of engagement.

The findings support further development of personalised perioperative information support. Refinement, assessment-item development, co-design and evaluation are needed before implementation. The current contribution is a transparent starting point for future nursing-led education interventions, confined to adult patients and not directly transferable to caregivers of children.

## Declarations

Manuscript IJNSA-D-26-00958

Information needs and engagement profiles of adults undergoing ambulatory surgery: a qualitative study

## Ethics approval and consent to participate

The study was approved by the hospital ethics committee of the participating institution (approval 2023-K172-01). All participants provided written informed consent before data collection, including consent to the anonymous reporting of quotations. They were informed that withdrawal would not affect their clinical care.

## Consent for publication

No identifiable participant images or personal details are published. Fig. 1 contains fictional characters and scenario dialogue; it does not depict research participants. Consent to the use of anonymous quotations is reported in the original study declaration.

## Declaration of generative AI and AI-assisted technologies in the manuscript preparation process

The interactions depicted in the illustration were fictionalised using an AI tool, with photographs of real-world settings serving as visual references. The illustration contains no photographs of study participants or identifiable patient data. These tools were not used to collect or analyse the interview data. The authors retain responsibility for the accuracy, integrity and final approval of the manuscript, translated quotations and illustrations.

## Funding

This research was supported by the Social Livelihood Science and Technology Fund of Nantong (grant MSZ2025128); Nantong Municipal Health Commission Research Project (grant MS2024007); and Affiliated Hospital of Nantong University Research Projects (grant Tfh 2619).

## CRediT authorship contribution statement

**Wen Qin:** Writing – review & editing, Writing – original draft, Supervision, Resources, Project administration, Methodology, Investigation, Data curation, Conceptualization. **Qin Zhu:** Writing – review & editing, Supervision, Resources, Project administration, Formal analysis, Conceptualization. **Yang Shen:** Software, Resources, Methodology, Investigation, Data curation. **Xiaoyun Dai:** Supervision, Resources, Methodology, Investigation, Formal analysis, Data curation. **Yi Xu:** Software, Methodology, Investigation, Data curation. **Yingying Huang:** Software, Investigation, Data curation. **Yao Tian:** Methodology, Investigation, Data curation.

## Declaration of competing interest

The authors declare that they have no known competing financial interests or personal relationships that could have appeared to influence the work reported in this paper.

## Data Availability

De-identified transcripts and supporting data are available from the corresponding author upon reasonable request, subject to institutional ethics approval and applicable data protection requirements. They are not publicly available to protect participant privacy.

## References

[bib0001] Alacadag M., Cilingir D. (2018). Presurgery anxiety and day surgery patients' need for information. J. Perianesth. Nurs..

[bib0002] Bailey C.R., Ahuja M., Bartholomew K., Bew S., Forbes L., Lipp A., Montgomery J., Russon K., Potparic O., Stocker M. (2019). Guidelines for day-case surgery 2019′. Anaesthesia.

[bib0003] Bandura A. (2004). Health promotion by social cognitive means. Health Educ. Behav..

[bib0004] Bhattacharyya O., Mossman K., Gustafsson L., Schneider E.C. (2019). Using human-centered design to build a digital health advisor for patients with complex needs: persona and prototype development. J. Med. Internet Res..

[bib0005] Braun V., Clarke V. (2006). Using thematic analysis in psychology. Qual. Res. Psychol..

[bib0006] Deng X., Liang S., Li H., Gouda D., Zhu T., Xiao K. (2019). A cross-sectional study to assess the difference in perception of day surgery information between patients and medical staff in China. Patient Prefer. Adherence.

[bib0007] Husain L., Finlay T., Husain A., Wherton J., Hughes G., Greenhalgh T. (2024). Developing user personas to capture intersecting dimensions of disadvantage in older patients who are marginalised: a qualitative study. Br. J. Gen. Pract..

[bib0008] Kim H., Xie B. (2017). Health literacy in the eHealth era: a systematic review of the literature. Patient Educ. Couns..

[bib0009] Malterud K., Siersma V.D., Guassora A.D. (2016). Sample size in qualitative interview studies: guided by information power. Qual. Health Res..

[bib0010] Miller S.M. (1987). Monitoring and blunting: validation of a questionnaire to assess styles of information seeking under threat. J. Pers. Soc. Psychol..

[bib0011] Ng J.Y.Y., Ntoumanis N., Thøgersen-Ntoumani C., Deci E.L., Ryan R.M., Duda J.L., Williams G.C. (2012). Self-determination theory applied to health contexts: a meta-analysis. Perspect. Psychol. Sci..

[bib0012] O'Brien B.C., Harris I.B., Beckman T.J., Reed D.A., Cook D.A. (2014). Standards for reporting qualitative research: a synthesis of recommendations. Acad. Med..

[bib0013] Perego F., Zingale L.C., Cesoni Marcelli A., Ranucci L., Rimoldi L., Nsanbayeva N., Natale M.R., Dalla Vecchia L.A., Gorini A. (2024). A human centred innovative approach based on persona in hereditary angioedema. Orphanet J. Rare Dis..

[bib0014] Rajala M., Kääriäinen M., Tanhua A., Kaakinen P. (2025). Patients' perspectives on postoperative follow-up calls in day surgery: a qualitative study. Scand. J. Caring Sci..

[bib0015] Sandelowski M. (2000). Whatever happened to qualitative description?. Res. Nurs. Health.

[bib0016] Stolk-Vos A.C., van der Steen J.J.E., Drossaert C.H.C., Braakman-Jansen A., Zijlmans B.L.M., Kranenburg L.W., de Korne D.F. (2018). A digital patient-led hospital checklist for enhancing safety in cataract surgery: qualitative study. JMIR Perioper. Med..

[bib0017] Street R.L., Makoul G., Arora N.K., Epstein R.M. (2009). How does communication heal? Pathways linking clinician-patient communication to health outcomes'. Patient Educ. Couns..

[bib0018] Thoen C.W., Sæle M., Strandberg R.B., Eide P.H., Kinn L.G. (2024). Patients’ experiences of day surgery and recovery: a meta-ethnography. Nurs. Open.

[bib0019] Vosbergen S., Mulder-Wiggers J.M.R., Lacroix J.P., Kemps H.M.C., Kraaijenhagen R.A., Jaspers M.W.M., Peek N. (2015). Using personas to tailor educational messages to the preferences of coronary heart disease patients. J. Biomed. Inf..

[bib0020] Wongkietkachorn A., Wongkietkachorn N., Rhunsiri P. (2018). Preoperative needs-based education to reduce anxiety, increase satisfaction, and decrease time spent in day surgery: a randomized controlled trial. World J. Surg..

[bib0021] Yang Z., Xu L., Gao Y., Zhang C., Wang A. (2025). Tailored personas for self-management in home-based cardiac rehabilitation for patients with coronary heart disease: a qualitative study. Int. J. Nurs. Stud..

[bib0022] Zimmermann M., Yonkers K.A., Tabb K.M., Schaefer A., Peacock-Chambers E., Clare C.A., Boudreaux E.D., Lemon S.C., Byatt N., Tulu B. (2024). Developing personas to inform the design of digital interventions for perinatal mental health. JAMIA Open.

