# Supplementary material for: Information needs and engagement profiles of adults undergoing ambulatory surgery: a qualitative study
Source: Int J Nurs Stud Adv. 2026 Oct 9;11:100703. doi: 10.1016/j.ijnsa.2026.100703 (PMC13697286; doi:10.1016/j.ijnsa.2026.100703)
Supplement: Supplementary file 1 [file mmc1.docx]

Supplementary file A

# Interview topics and example questions reported in the study

This supplement reproduces the topics and example questions reported in the manuscript. It is not presented as the complete original interview guide. The source describes development through a focused literature review, consultation with four clinical experts and pilot interviews with two patients.

| **Topic** | **Example question** |
| --- | --- |
| Decision-making before admission | What were your main questions when you first decided to have surgery? |
| Waiting for surgery | While waiting for surgery, what information did you feel was missing? |
| Discharge and care at home | What are you most uncertain about regarding care at home? |
| Preferred information delivery | How would you ideally like to receive this information? |

# Examples of follow-up prompts

Could you tell me more?

What do you mean by “rest”?

# Administration described in the manuscript

Individual face-to-face interviews were conducted in a private consultation room, lasted 30–60 minutes and were audio-recorded with consent. Field notes were written immediately afterwards. Mandarin transcripts were checked against the recordings and de-identified. The current analysis includes adult patients only.
